# Case report: Drug rash with eosinophilia and systemic symptoms syndrome in a patient with anti–interferon-γ autoantibody–associated immunodeficiency

**DOI:** 10.3389/fimmu.2022.969912

**Published:** 2022-08-22

**Authors:** Yuxue Nie, Han Wang, Xiying Dong, Siqi Pan, Ting Zhang, Jun Ran, Ying Zhang, Junping Fan, Linqi Zhang, Jinglan Wang

**Affiliations:** ^1^ Department of Pulmonary and Critical Care Medicine, Peking Union Medical College Hospital, Chinese Academy of Medical Sciences (CAMS) and Peking Union Medical College (PUMC), Beijing, China; ^2^ Comprehensive AIDS Research Center, Center for Infectious Diseases Research, Beijing Advanced Innovation Center for Structural Biology, School of Medicine, Tsinghua University, Beijing, China; ^3^ School of Clinical Medicine, Chinese Academy of Medical Sciences (CAMS) and Peking Union Medical College (PUMC), Beijing, China; ^4^ Heart Failure Center, Fuwai Hospital, National Center for Cardiovascular Diseases, Chinese Academy of Medical Sciences (CAMS) and Peking Union Medical College (PUMC), Beijing, China; ^5^ Department of International Medical Service, Peking Union Medical College Hospital, Chinese Academy of Medical Sciences (CAMS) and Peking Union Medical College (PUMC), Beijing, China

**Keywords:** adult-onset immunodeficiency due to anti-interferon-gamma antibodies, anti-IFN-γ autoantibodies, infection, sulfamethoxazole, *Burkholderia*, drug rash with eosinophilia and systemic symptoms

## Abstract

A 56-year-old Chinese woman with previous disseminated mycobacterium avium complex infection and recurrent cervical abscesses from *Burkholderia cepacia complex* visited our hospital. She was diagnosed with adult-onset immunodeficiency (AOID) and tested positive for interferon-γ–neutralizing autoantibody. Ceftazidime was administered as the initial antimicrobial treatment, which was later combined with sulfamethoxazole-trimethoprim (SMZ-TMP). She developed drug rash with eosinophilia and systemic symptoms (DRESS) syndrome after SMZ-TMP administration and improved after withdrawal of the culprit antibiotic and systemic glucocorticoids treatment. Her cervical infection was eventually cured after combined therapy of long-term antibiotics and anti–IFN-γ autoantibodies (AIGA) titer-lowering treatments including glucocorticoids, rituximab, and plasmapheresis. This is the first case of DRESS syndrome in the setting of AIGA-induced AOID and is worthy of notice.

## Introduction

Adult-onset immunodeficiency (AOID) caused by anti–interferon-γ (IFN-γ) autoantibodies (AIGAs) is an autoimmune disorder characterized by a defect in the IFN-γ pathway, making the host susceptible to infections by various intracellular pathogens [e.g., tuberculosis and non-tuberculous mycobacterium (NTM) species, *Burkholderia*, and *Talaromyces*] (1, 2). AOID’s treatment regimen is composed of both the clearance of AIGAs and antimicrobial agents when complicated by infection. Drug rash with eosinophilia and systemic symptoms (DRESS) syndrome is a rare, potentially life-threatening drug-induced reaction (3). Antibiotics, such as sulfamethoxazole-trimethoprim (SMZ-TMP) and minocycline, are common causes of DRESS (4). Herein, we report a rare case of patient with AOID who had cervical abscesses from *Burkholderia cepacia complex* (*BCC*) infection and later developed DRESS syndrome after SMZ-TMP administration. This case report follows the CARE guidelines (5).

## Case report

A 56-year-old woman from Fujian, China, presented to our hospital with a 10-month history of cervical abscesses in October 2020. She had been diagnosed with disseminated mycobacterium avium complex (MAC) infection that involved the lungs, breasts, skin, lymph nodes, and spine 20 months prior to admission. Since then, she had been put on antimycobacterial therapy with rifampicin (450 mg q.d.), ethambutol (750 mg q.d.), clarithromycin (500 mg b.i.d.), levofloxacin (500 mg q.d.), and minocycline (100 mg b.i.d.). Her first abscess was found on the right neck in 2019, repeatedly cultured positive for *BCC*. It took 4 months for the abscess to resolve by intravenous ceftazidime. The patient developed another abscess on the left side of her neck 1 week prior to this admission. She was still taking anti-mycobacterial regimen (rifampicin, ethambutol, and clarithromycin) upon admission. Her previous history was notable for herpes zoster. She reported no history of allergy.

**Figure 1:**
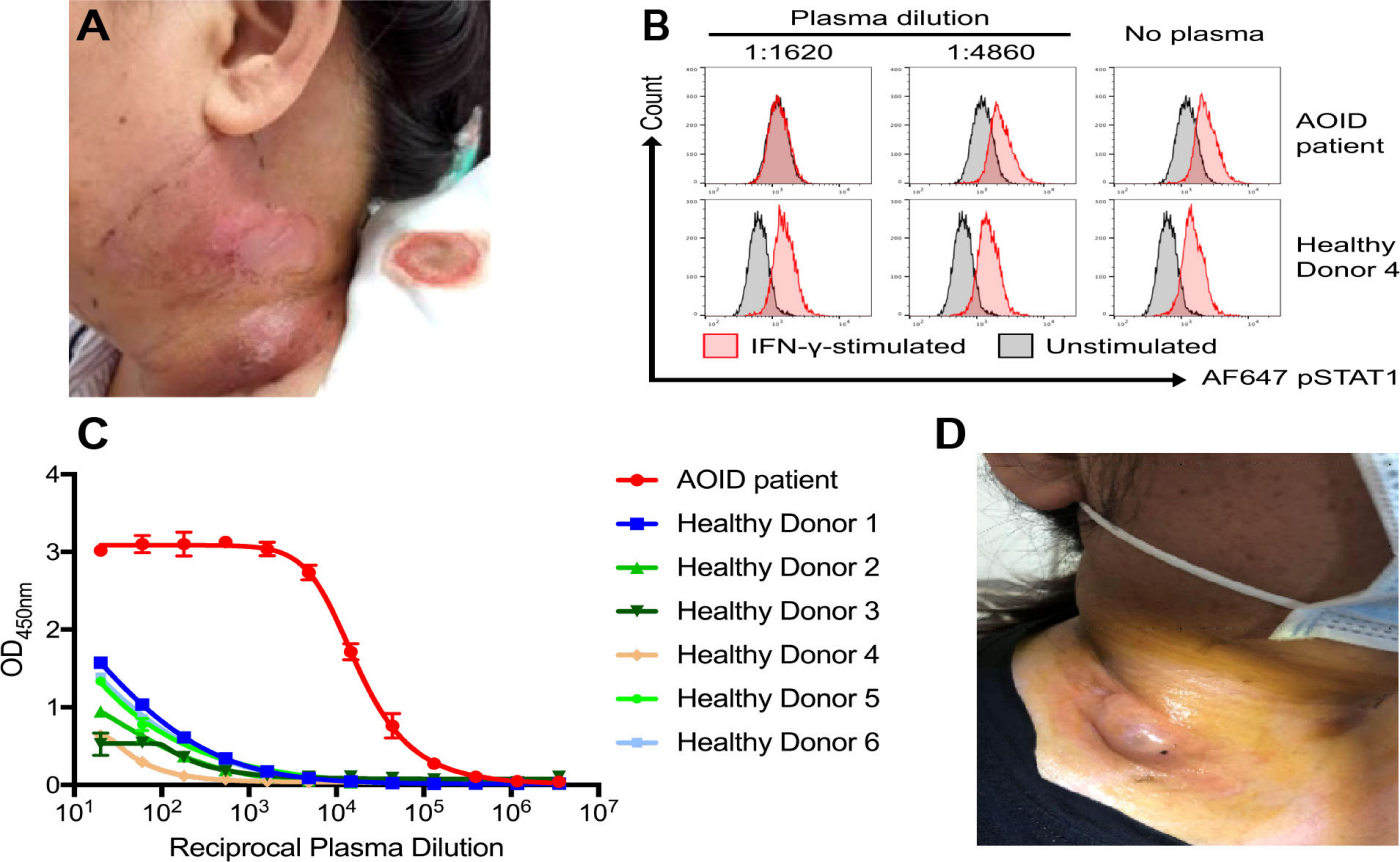
**(A)** The cervical abscess at admission. **(B)** STAT1 phosphorylation in THP-1 cells was inhibited when incubated with plasma sample from the patient at 1:1,620 dilution but remained consistent with samples from healthy donors. Representation of two independent experiments was shown. **(C)** Anti–IFN-γ IgG were detected in the patient’s plasma using ELISA at depicted dilutions. STAT1, signal transducer and activator of transcription 1; IFN-γ, interferon-γ; ELISA, enzyme-linked immunosorbent assay. **(D)** The deterioration of cervical abscess during treatment.

On admission, her temperature was 36°C, blood pressure was 137/83 mm Hg, heart rate was 85 per minute, respiratory rate was 18 per minute, and oxygen saturation was 96% on room air. There were two masses on the left side of her neck with tenderness on palpation (**Figure 1A**). There was no lymphadenopathy or hepatosplenomegaly on physical examination. Laboratory findings were listed in **Table 1** and showed elevated erythrocyte sedimentation rate (ESR): 96 mm/h (0–15 mm/h), high level of C-reactive protein (CRP): 56 mg/L (0–8 mg/L), and high levels of immunoglobin G (IgG) and IgM: 32.79 g/L (7–17 g/L) and 2.67 g/L (0.40–2.30 g/L), respectively. The test for cytomegalovirus by quantitative polymerase chain reaction was negative. The human immunodeficiency virus serology test was negative. The CD4^+^ T-cell count was 484 per mm^3^ (561–1,137 per mm^3^). Metagenomic next-generation tests for primary immunodeficiency were negative. Culture of the pus extracted from the cervical abscesses was negative for MAC and positive for *BCC*, which was sensitive to ceftazidime and SMZ-TMP. Considering the patient had been repeatedly infected by opportunistic intracellular pathogens, we evaluated AIGAs in the patient’s plasma using enzyme-linked immunosorbent assay (ELISA). A high titer of AIGA IgG was detected in the patient’s plasma with a half-maximum titer of 13,363 (normal range < 20). Furthermore, her AIGAs were capable of inhibiting IFN-γ signaling when tested by IFN-γ–induced STAT1 phosphorylation of human monocyte-like cell line THP-1 cells (**Figures 1B, C**), and AOID was diagnosed. The details of the test can be found in the **Supplementary materials**.

**Table 1:** Laboratory data.

Variable	Reference Range, Adults, This Hospital*	On This Admission	Symptom Onset of DRESS (Hospital Day 37)	On This Discharge (Hospital Day 216)
Hemoglobin (g/dl)	11–15	10.9	11.4	13.2
Hematocrit (%)	35.0–50.0	33.2	33.3	39.0
White-cell count (per mm^3^)	3,500–9,500	10,730	19,160	6,710
Differential count (per mm^3^)				
Neutrophils	2,000–7, 500	9,227	16,937	5,247
Lymphocytes	800–4, 000	1,255	823	845
Monocytes	120–800	515	344	503
Eosinophils	20–500	319	1,034	20
Platelet count (per mm^3^)	100–350	229	/	112
Alanine aminotransferase (U/L)	7–40	11	21	47
Aspartate aminotransferase (U/L)	13–35	10	19	17
Total bilirubin (mg/dl)	0–1.3	0.30	0.33	0.86
Direct bilirubin (mg/dl)	0–0.4	0.10	0.20	0.19
Carbon dioxide (mmol/L)	20.0–34.0	28.9	22.3	31.8
Urea (mmol/L)	2.78–7.14	5.51	6.49	6.25
Creatinine (mg/dl)	0.51–0.95	0.87	1.44	0.48
Erythrocyte sedimentation rate (mm/h)	0–20	108	87	6
C-reactive protein (mg/L)	0–8	56	126	0.2
Immunoglobin G (g/L)	7.00–17.00	32.79	/	8.93
Immunoglobin A (g/L)	0.70–4.00	3.89	/	2.00
Immunoglobin M (g/L)	0.40–2.30	2.67	/	1.33
CD4^+^ T-cell count (per mm^3^)	561–1,137	484	/	223
CD8^+^ T-cell count (per mm^3^)	404–754	554	/	434

*Reference values are affected by many variables, including the patient population and the laboratory methods used. The ranges used at Peking Union Medical Hospital are for adults who are not pregnant and do not have medical conditions that could affect the results. They may therefore not be appropriate for all patients.

**Figure 2:**
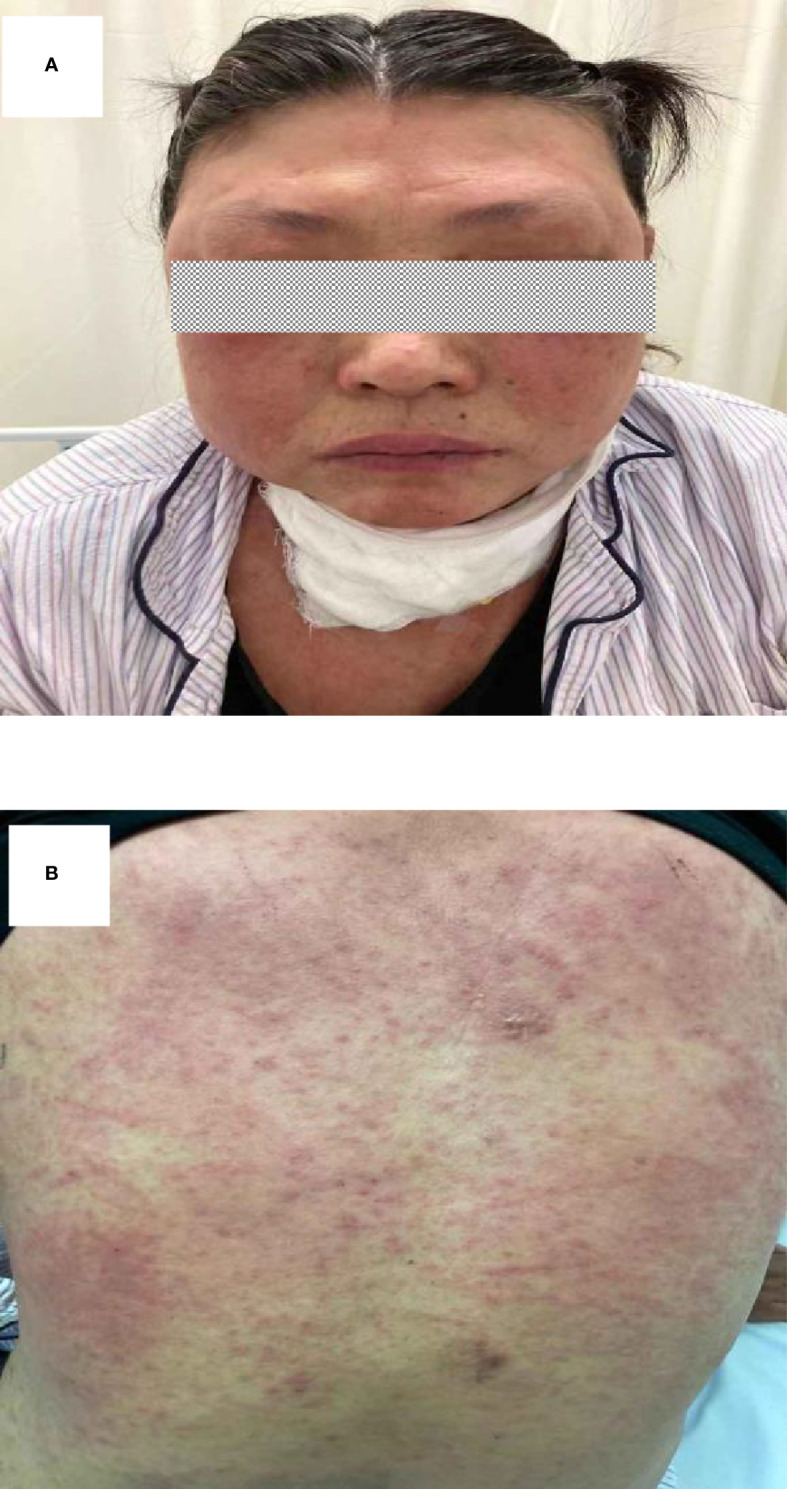
**(A, B)** Rash that developed 18 days after the first administration of SMZ-TMP. SMZ-TMP, sulfamethoxazole-trimethoprim.

To treat the infection of BCC, intravenous ceftazidime (1,000 mg t.i.d.) was used in this patient. However, her abscess deteriorated despite the ceftazidime treatment (**Figure 1D**). Given the patient’s immunodeficiency and the clinical deterioration of abscess with ceftazidime alone, oral SMZ-TMP (160 mg/800 mg, two tablets b.i.d.) was added. Unexpectedly, 18 days from the start of SMZ-TMP treatment, she developed fever (39°C), prominent facial and palmoplantar edema, and polymorphous maculopapular eruption [>50% of body surface area (BSA)] (**Figures 2A, B**). In the following days, she experienced oliguria and was diagnosed with acute kidney injury. Her lab tests were notable for a white blood cell count of 19.16 × 10^9^/L, peripheral eosinophilia (9.34%), atypical lymphocytes on peripheral smear, and elevated creatine level of 1.44 mg/dl (0.51–0.95 mg/dl). Blood culture, serology of hepatitis viruses (HAV, HBV, and HCV), and antinuclear antibody were all negative. DRESS syndrome was confirmed according to the International Registry of Severe Cutaneous Adverse Reactions (RegiSCAR) system. Considering the time of SMZ-TMP use and the onset of DRESS syndrome, SMZ-TMP was considered as the culprit drug. We did not perform patch testing or intradermal testing on the patient due to the severe situation at that time. As a result, we stopped her SMZ-TMP but continued other antibiotics (ceftazidime and antimycobacterial agents) and started glucocorticoids (intravenous methylprednisolone, 40 mg b.i.d.) and intravenous immunoglobulin to treat DRESS syndrome. The rash improved gradually over the next month. It was important to mention that her cervical abscesses surprisingly improved under the strong immunosuppression by glucocorticoids.

**Figure 3:**
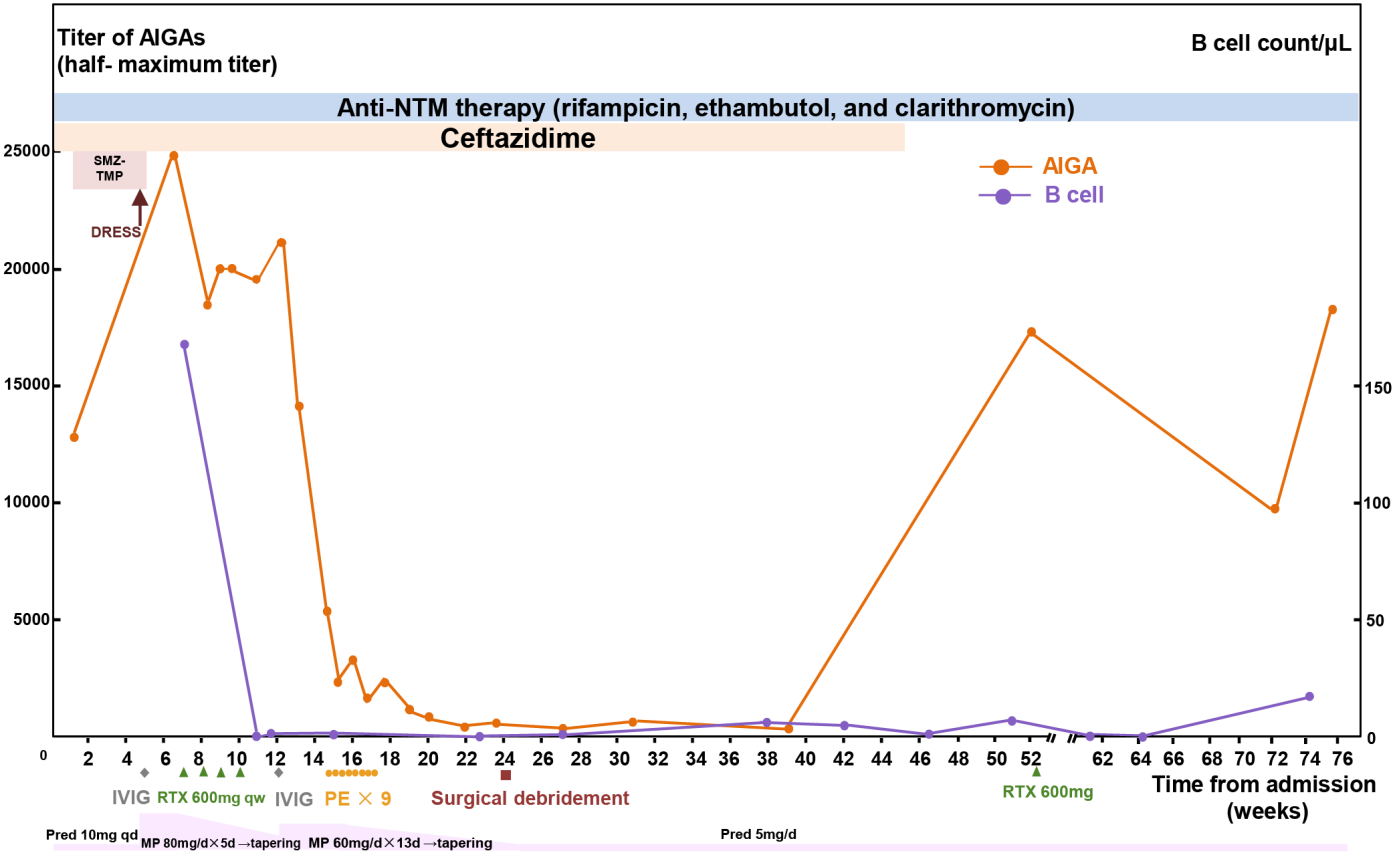
Summary of clinical course. The concentrations of half-maximum titers of AIGAs and circulating B-cell counts during the process of treatment. AIGA, anti–interferon-γ autoantibodies; MP, methylprednisolone; Pred, prednisone; DRESS, drug reaction with eosinophilia and systemic symptoms; SMZ-TMP, sulfamethoxazole-trimethoprim; NTM, non-tuberculous mycobacteria; RTX, rituximab; IVIG, intravenous immunoglobulin; PE, plasma exchange.

Moreover, the patient received B-cell–depleting therapy with rituximab (375 mg/m^2^ on d1, d8, d15, and d22) and plasmapheresis to treat the AOID after informed consent. AIGA titer decreased to a lower level, and her abscess improved after these treatments (**Figure 3**). She also received surgical debridement of the abscesses 24 weeks after admission. The AIGA titers, B-cell counts, and respective treatments over time were summarized in **Figure 3**. Her abscesses improved gradually and healed 3 months after the debridement. Ceftazidime was administered for 45 weeks in total (39 weeks after discontinuation of SMZ-TMP). No relapse of either abscesses or DRESS syndrome had been observed in more than 1 year, although the levels of CRP (33 mg/L), ESR (58 mm/h), and AIGA titer (18,046) silently rebounded (**Figure 3**).

## Discussion

The diagnosis of AOID should be considered in individuals suffering from disseminated mycobacterial or other opportunistic infections without known immunosuppression. The diagnosis requires the detection of AIGA and assessment of its neutralizing activity against IFN-γ. DRESS syndrome is a rare hypersensitivity drug reaction, which usually begins 2–6 weeks after the inciting drug exposure and often manifests with fever, widespread skin lesions, eosinophilia, and end organ damage. This patient acquired six points (one point for fever, one point for eosinophilia, one point for atypical lymphocytes, one point for skin rash >50% BSA, one point for skin rash suggesting DRESS, one point for renal involvement, one point for exclusion of other causes, and minus one point for the resolution less than 15 days) by the RegiSCAR criteria and was classified as a “definite case” for DRESS syndrome. SMZ-TMP was probably the inciting drug as evidenced by the time of onset, effective symptom relief after its withdrawal, and previous reports of SMZ-TMP–induced DRESS cases.

To the best of our knowledge, there have been no reports of DRESS syndrome in patients with AOID. Because of the defect in the host’s immune system to eliminate the offenders, the antimicrobial treatment of intracellular pathogens in AOID usually calls for a combined and prolonged course of antibiotics. DRESS syndrome is a potentially fatal drug reaction, with a mortality of 2%–10% (4, 6). As to its offending agents, antibiotics constitute an unneglectable part, which include antituberculosis medications (rifampin, isoniazid, ethambutol, and pyrazinamide), minocycline, SMZ-TMP, and vancomycin (7). There are also reports on macrolides, fluoroquinolones, and cephalosporins (7). Moreover, it is notable that the use of high-dose glucocorticoids for DRESS syndrome in this patient did not exacerbate the cervical abscess. Therefore, a comorbidity of active infection in AOID should not be a restraint to glucocorticoids’ use when DRESS syndrome occurs.

The potential correlation between the AIGAs and DRESS is of interest. First of all, because IFN-γ is important in the polarization and activation of M1 macrophages to clear the intracellular pathogens, this process can inhibit type 2 immune response. Intriguingly, it is known that polarization toward type 2 immune response is pivotal in the pathogenesis of DRESS syndrome (8). Therefore, we speculate that the presence of AIGAs can limit the suppression of IFN-γ on type 2 immune response to some extent, thus making the host predisposed to DRESS in this circumstance. There is an association between HLA-DRB1*15:02–HLA-DQB1*05:01 and AIGAs in Southeast Asian patients (9). For drug hypersensitivity reactions caused by SMZ-TMP, HLA-B∗13:01 was associated with DRESS syndrome in Asians (10), and HLA*B15*02 was associated with Stevens–Johnson syndrome/toxic epidermal necrolysis (11). HLA genotype may be a possible explanation for the co-occurrence of AOID and DRESS in this case. However, HLA genotype was not tested in this case; thus, this speculation is presumable. Because AOID has only been recognized in recent years, the potential connection between AOID and DRESS syndrome remains to be explored.

## Conclusion

Our case indicates that DRESS syndrome can develop in patients with AOID. Immunosuppressive treatment for DRESS syndrome did not exacerbate the concurrent infection. Further study is expected to investigate the potential association between AOID and DRESS.

## Data availability statement

The original contributions presented in the study are included in the article/**Supplementary Material**. Further inquiries can be directed to the corresponding author.

## Ethics statement

Written informed consent was obtained from the individual(s) for the publication of any potentially identifiable images or data included in this article.

## Author contributions

YN, SP, TZ, and JR collected and interpreted the patient data. HW performed the ELISA and neutralizing tests; all abovementioned authors participated in manuscript writing. YZ and XD participated in writing and editing the manuscript. JF and JW conceived this study and coordinated the author group. All authors have read and approved the final manuscript.

## Funding

CAMS Innovation Fund for Medical Sciences (CIFMS) 2021-I2M-1-048. This funding source had no role in the design of this study and will not have any role during its execution, analyses, interpretation of the data, or decision to submit results.

## Acknowledgments

We would like to thank the patient for allow publication of the case details.

## Conflict of interest

The authors declare that the research was conducted in the absence of any commercial or financial relationships that could be construed as a potential conflict of interest.

## Publisher’s note

All claims expressed in this article are solely those of the authors and do not necessarily represent those of their affiliated organizations, or those of the publisher, the editors and the reviewers. Any product that may be evaluated in this article, or claim that may be made by its manufacturer, is not guaranteed or endorsed by the publisher.
