# Supplementary material for: Case report: Drug rash with eosinophilia and systemic symptoms syndrome in a patient with anti–interferon-γ autoantibody–associated immunodeficiency
Source: Front Immunol. 2022 Aug 22;13:969912. doi: 10.3389/fimmu.2022.969912 (PMC9441898; doi:10.3389/fimmu.2022.969912)
Supplement: Supplementary file 1 [file DataSheet_1.docx]

Supplementary Material

# Supplementary Data

The methods for detection of the anti-interferon-γ autoantibodies and the evaluation of its inhibition effect

**ELISA**

96-well ELISA plates (Corning) were coated with 1 μg/ml of Recombinant Human IFN-γ Protein (R&D, 285-IF/CF) in PBS (Gibco) overnight at 4 °C. Plates were washed 5 times between each steps with PBS containing 0.05% Tween-20. Coated plates were blocked with PBS containing 5% bovine serum albumin for 1 h at 37 °C. Serially diluted plasma samples of the patient and healthy donors (starting at 1:20 dilution) were added to the plates and incubated for 1 h at 37 °C. Plates were then incubated with anti-human IgG conjugated with horseradish peroxidase (HRP) (Promega, W403B) at 1:5,000 dilution for 30 min at 37 °C and further developed using 3,3’,5,5’-tetramethylbenzidine (TMB) (CWBio). The reaction was stopped by adding 1M H_2_SO_4_ and absorbance was measured at 450 nm with an iMark Microplate Absorbance Reader (Bio-Rad). Half-maximum titers were calculated using the dose-response model with asymmetric five-parameter equation in GraphPad Prism 7 (GraphPad Software).

**Evaluation of IFN-γ-induced STAT1 phosphorylation by flow cytometry**

Four thousand U/ml of Human IFN-γ Recombinant Protein (final concentration 1,000 U/ml) in 50 μl of RPMI-1640 (Gibco) was incubated with 50 μl of threefold serially diluted plasma samples either from the patient or from healthy donors (starting at 1:60 dilution) at 37 °C for 1 h before adding to 10^5^ THP-1 cells (ATCC, TIB-202) in 100 μl of RPMI-1640. After incubation for a further 15 min at 37 °C, cells were fixed by adding 200 μl of Cytofix/Cytoperm Fixation/Permeabilization Solution (BD Biosciences) at 4 °C for 20 min. Cells were then washed twice in PBS containing 2% FBS and permeabilized with 300 μl of ice-cold absolute methanol on ice for 15 min. After two washing steps, Alexa Fluor 647 phospho-STAT1 (pY701) antibody (BD Phosflow, 612597) was added at a dilution of 1:50 and incubated at 4 °C for 30 min, washed twice and resuspended in PBS containing 2% FBS. Data were collected with a BD LSRFortessa flow cytometer (BD Biosciences) and analyzed with FlowJo (BD Biosciences).
